# A descriptive study of chiropractors' opinions and practices regarding office-based health product sales

**DOI:** 10.1186/2045-709X-20-10

**Published:** 2012-04-05

**Authors:** Stacey A Page, Jaroslaw P Grod, D Gordon McMorland

**Affiliations:** 1 Office of Medical Bioethics, RM 93, HMRB, Faculty of Medicine, University of Calgary, 3330 Hospital Drive NW, Calgary AB. T2N-4N1, Canada; 2 Private practice, Toronto, ON; 3 National Spine Care, #300 - 301 14th Street NW, Calgary, Alberta T2N 2A1, Canada

**Keywords:** Chiropractic, Professional ethics, Codes of ethics, Marketing

## Abstract

**Background:**

Although the sale of non-prescription health products is ubiquitous, the views of health professionals, such as chiropractors, regarding the sale of such products are not well known. Practitioner opinion is important to understand and inform professional practice. The purpose of this study was to describe chiropractors' perspectives and practices on the sale of health care products from practitioners' offices.

**Methods:**

Chiropractors were invited to provide written comments about health product sales at the end of a fixed choice, mailed survey. Respondents' comments were analyzed using qualitative description. Ethics approval was received from the Conjoint Health Research Ethics Board at the University of Calgary.

**Results:**

One hundred seven of the 265 respondents (response rate of 51%) provided written comments. Approximately 30 pages of double-spaced, typed text were gathered. Respondents did not consistently endorse or condemn health product sales, and engaged in the practice to greater and lesser extents. While some were opposed to health products sales, some accepted the practice with a degree of ambivalence whereas others clearly embraced it. Some respondents acknowledged a professional conflict of interest in such sales and marketing, and described strategies used to mitigate it. Others provided a range of justifications for the practice. Personal integrity and professional standards were discussed and a need for monitoring identified.

**Conclusions:**

A wide range of opinions and practices were described and this is consistent with resulting variation in practice. In light of this, standards that facilitate consistency in practice may benefit professionals and the public alike.

## Background

The sale of non-prescription health products is ubiquitous, meeting a significant consumer demand [1,2]. Natural health products, such as vitamins, minerals, herbal remedies, homeopathic and traditional medicines, nutritional supplements, and products such as cosmeceuticals, rehabilitation and fitness aids can be found online, in major retail chains, mall kiosks and in the offices of health professionals. A recent report estimated the total retail sales of natural health products in Canada to be worth $2.5 billion dollars in 2005, growing to an estimated $2.75 billion by 2010 [1]. Distributors identified healthcare practitioners as among their top three current and future most important distribution channels [1]. The regulation of and evidence base for many of these products are uneven or non-existent, leaving consumers to fend for themselves in a "buyer beware" market [3-5].

The roles and responsibilities of health professionals regarding the sale of non-prescription health products are unclear [5-7]. Should health professionals be marketing health goods to their patients? If so, to what standards should they adhere? Should there be any limits placed upon this practice? How are such sales monitored by professional bodies?

Research considering the sale of health products by health professional is limited. There have been a few studies that have explored the practice among dermatologists [7,8], dentists [9] and most recently chiropractors [6,10]. Together, these studies suggest product retailing by health professionals is prevalent. Proponents believe that it affords control over product quality, that it is a service patients appreciate, and that treatment compliance is enhanced [11]. Opponents argue that product sales are undertaken for profit, thus presenting a conflict of interest compromising the therapeutic relationship and professional integrity [12-14].

Considering chiropractic specifically, studies have suggested that the majority of practitioners (54-89%) sell a range of products [6,10,15]. Chiropractic trade reports and publications suggest product sales are a major revenue source, improving practice profitability for chiropractors [16-18].

Relative to other disciplines, the public trust in the healing professions is high [19]. Such trust is dependent upon practioners upholding their duties to act in their patients' best interests and is central to the effectiveness of the patient-practitioner relationship. Professional practice is guided in part by codes of ethics and conduct. As the health product market place continues to grow, so too will the potential for ethical conflicts faced by practitioners choosing to retail health products. Little is known about chiropractors' individual considerations of this practice

This study reports on the qualitative findings of a previous [10] survey where chiropractors had the opportunity to comment on the sale of health products. Guidance offered by the Alberta Chiropractic Association and College and the Canadian Chiropractic College are considered.

## Methods

A random sample of 518 chiropractors (of 929 total population) in the province of Alberta, Canada received an initial survey package with a follow-up package sent out three weeks later., The four-page survey contained 34 fixed-choice questions about chiropractors opinions and practices surrounding health product sales. Subjects were invited to provide written comments about the sale of health products by chiropractors. Subjects were assured their data would be de-identified. The confidentiality of study responses was preserved by replacing respondents' names with codes and be reporting all findings in aggregate. This study received ethics approval from the Conjoint Health Research Ethics Board at the University of Calgary.

## Results

Two hundred sixty five chiropractors (51.2%) completed and returned the survey. Respondents were mostly men (N = 207; 78.1%) and had been in practice for a median of 12 years (IQR 8-20 years). Most were in multidisciplinary group practices (N = 204; 77.3%) and most (N = 208; 78.5%) were satisfied with their number of weekly patient contact hours. The majority (N = 247; 95.0%) believed that chiropractors should sell health products and 89.4% (N = 236) currently sold health products from their practices. The quantitative survey results are presented elsewhere [10]. This paper reports on the analysis of the volunteered, qualitative comments.

The written comments of 107 respondents were transcribed, yielding approximately 30 pages of double-spaced typed text. The text was entered into the Ethnograph (qualitative analytic) software [20]. The first author (SP) reviewed, coded and summarized the textual data using qualitative description. Qualitative description is a low-inference interpretation that focuses on describing "the facts [21]". codes and summary was reviewed against the raw data by the two other authors (JG, GM). No discrepancies in interpretation occurred.

The following themes emerged.

### Conflict of interest

Several commentators readily acknowledged the potential for a conflict of interest to exist when treating chiropractors were also involved in health product sales. Respondents spoke generally about the potential for conflict of interest to occur when any health care practitioner "over treats" a patient.

Some respondents were very clear in their opinion that product sales crossed an ethical line *(2-165 They are unethical and inappropriate when they are central to revenue generating strategies)*. Several were aware of instances in which chiropractors appeared to push products sales *(*e.g.*, 2-149 "I have some concern with practices which profess to be strongly "anti-allopathic" against the "pill and a bill" practices of medicine yet you always leave the clinic with a bag of supplements to assist this and prevent that;" 2-187 "The concern I have is when it is dispensed/promoted as a profit centre. This has created lots of bad will when patients are encouraged to buy bags of products...")*

Others held a more ambivalent position, where the motivations toward practitioner profit or patient well being were sometimes described as "hard to distinguish. Some likened keeping these interests in balance as a fine line or something that one had to be constantly mindful of (2-220), while others admitted feeling uncomfortable about engaging in the practice *(*e.g.*, 2-149 "I try to satisfy my guilt for carrying this type of stuff by having a low margin")*.

Finally, some respondents experienced no conflict of interest at all and felt that health product sales fit well within their practices. (e.g., *"...The feedback I got from patients describing the knowledge base I had was greater than anyone they had met got me to realize the time and money spent on my education was not only valuable but that it was worth charging a full fee. I am exceptionally comfortable with this position.")*

### Mitigating actions

A second category of responses centred on practices or behaviours that respondents believed mitigated potential conflict of interest.

To this end, chiropractors described provisions to minimize perceived conflicts of interest. Most often, chiropractors talked about the mark-up they placed on products sold, attempting to minimize conflict of interest by selling products at cost or for a very small mark up. Others acknowledged a profit margin. The descriptions ranged from statements like the practitioner was "not out to make money (2-35)" to a "very modest mark-up" to a "small profit" to "fair market value." The labor costs associated with maintaining a product inventory, and for spending the time discussing such products with patients were also presented as justifications for how costs were structured (*2-21 "Keeping stock and dealing with GST is laborious...." 2-581 "It is time consuming to advise patients on why these products are helpful and to advise them on proper consumption levels *etc. *to advance their recovery. The mark-up on products 20-30% is poor compensation for the time"*).

Despite supporting sales generally, several respondents expressed their opposition to multi-level marketing.

A second initiative undertaken to alleviate the perception of competing interests was the efforts to ensure products were purchased voluntarily. Chiropractors used terms like '"don't hard sell" and "unpushy" to emphasize that purchases were at the patient's discretion. Some noted they directed patients to other places where such purchases could be made (*2-08 "When I make recommendations to patients re: products I always advise them that they don't need to purchase them from our clinic and I provide other potential sources")*. A few acknowledged they had observed aggressive marketing among their colleagues *(*e.g.*, 2-74 Some DCs are very pushy about selling products and thus make good money from it)*.

### Justifications

Another theme that emerged in the comments was how product sales were justified by the respondents. Several arguments were identified including professional role, scope of practice and quality assurance considerations.

Most commonly, respondents believed that product sales were consistent with their duty or obligation to act in patient's best interest. Products that promoted health or healing were viewed as enhancements to patient care and in line with the chiropractor's role *(*e.g.*, 2-219 "I feel I use health care products the same way I use my adjustments - that is for the good of the patient)*. Within this argument, a range of perspectives was again evident. While some viewed any contribution to health as a justification for product sales *(*e.g.*,2-339 In regards to supports (*e.g.*, in-soles, pillows) they are a mandatory prerequisite to patient health and if I do not carry them, I would be doing my patients a disservice) *others suggested that product sales should be based on clinical need and response to a presenting condition only.

Some chiropractors further argued that sales were well within their scope of practice and fields of expertise *(*e.g.*, 2-137 Chiropractors...receive extensive training in the appropriate use of those health products and as such have the right and responsibility to prescribe these products to patients if they would be of benefit)*. A few suggested that the training and resulting expertise that chiropractors had with respect to the use of health products generally, and nutritional products specifically, is far superior to other disciplines, in particular medicine.

Contrasting with these perspectives, some cautioned that chiropractors might not have the necessary expertise to support certain product sales *(*e.g.*, 2-103 I think some chiropractors overstep their boundaries and make nutritional supplements a huge part of their practice without proper educational back-up)*. Others believed that there were limits to what chiropractors should be offering to enhance patient well-being *(2-551 "As chiropractors do not take kindly to other practitioners doing spinal manipulation, I think it reflects poorly on our profession when we get excessively involved by areas better served by naturopaths (*e.g.*, supplements) and podiatrists (foot assessment and orthotics))*

Some practitioners asserted that they could attest to the quality of the products they sold, in contrast to the potentially unknown quality available on the general market. References were made to products being of "very good quality," or "best quality available."

Practitioners also spoke of having researched the products that they sold, with a few citing specific sources such as peer reviewed literature or manufacturers' standards.

Some believed that product sales were a convenience offered to patients and were justified on the basis of patient demand. Further to this, some chiropractors noted they only sold products available to health practitioners or that were otherwise hard to find or unavailable. A few suggested that having products readily available enhanced treatment compliance.

### Ethics and regulation

A group of respondents averred that professionalism and ethics in practice were a matter of individual integrity. If a chiropractor was ethical as an individual, there was no reason to doubt his or her integrity in professional practice. Similarly, respondents acknowledged that there are typically a few people in any professional group whose actions are not consistent with the values of the whole. Specifically considering product sales, respondents focused on the practitioner's intent, that is, where engaging in sales clearly for the patient's well being was acceptable whereas engaging in it for profit was not.

Finally, although individual integrity was noted to be critical in the maintenance of professionalism in practice, some spoke of the need for the profession to monitor individual practices and sanction those who clearly violated standards of practice. A need for guidelines, education and awareness for chiropractors relating to product sales was identified.

## Discussion

Chiropractors who responded to this survey did not uniformly support or condemn the practice of health product sale and, consistent with this engaged in product sales to varying extents. While a few chiropractors were implacably opposed to health product sales, most accepted the practice under various conditions and with varying degrees of comfort. Some clearly embraced the practice. Consistent with arguments in the literature, those engaged in the health product sales believed it was a convenience for patients and part of their role and responsibility as health care providers. Those who offered comments recognizing potential harms spoke of the practice as compromising professional integrity and described strategies to minimize negative effects.

Conflict of interest relates to a set of conditions or a situation such that professional judgment about a primary interest may be unduly influenced by a secondary interest [22]. Conflict of interest in selling health products from within a professional practice occurs if the potential sale of a product causes practitioners to deviate from their professional obligations to their patients in anticipation of economic gain. Assuming products are safe and efficacious, selling them is ethically neutral. Selling products primarily for profit rather than for patient necessity violates the practitioner's fiduciary duty to act in patient's best interests as their own interests prevail [12]

Recommended strategies to address conflict of interest involve taking steps to avoid or prevent the set of conditions provoking it [22]. Indeed, a few respondents stated that they did not engage in product sales or minimized the conflict by maintaining no/low profit margins.

Others emphasized that purchases were made voluntarily by patients, arguing that they had no influence over the patient's actions, a disingenuous claim at best in view of the nature of the therapeutic relationship and its inherent power imbalance. Patients may feel that purchases are necessary in order to maintain a positive relationship with the practitioner or to receive future care. Little research has investigated this area however one small study of patient opinion indicated that patients reason for purchasing products from practitioners was their trust in the practitioner [8]. Anecdotal reports suggest that such purchases may leave patients feeling exploited by practitioners [23]. While subtle influence may be pervasive, more worrisome is the allegation of aggressive sales strategies used by some of the respondents' colleagues.

Conflict of interest rules are a means of acknowledging that such problems may arise and offering guidance for individuals in managing these circumstances. Conflict of interest involving health product sales is recognized within the Canadian Chiropractic Association's (CCA) national code of ethics and conduct of chiropractors and also within Alberta College and Association of Chiropractors (ACAC), both of which permit health products sales provided certain conditions are met. The relevant section from the CCA's Code of Ethics and Conduct (Article 21) states:

It is not unethical to dispense items providing; it does not create a conflict of interest, they serve the best interests of the patient, clinical value has been demonstrated, and the item is available at a fair market price [24].

The Alberta College and Association of Chiropractors Code of Ethics provides the following guidelines:

A chiropractor who sells or markets professional products to their patients must:

• Ensure that they do not exploit the trust inherent in the chiropractor-patient relationship

• Not misrepresent or exaggerate the value of the products

• Prior to the sales of the product, have thoroughly evaluated the information related to the product and must be satisfied that the therapeutic value represented is rational

• Make available to patients all information necessary for the patients to make an informed choice as to whether to purchase the products, including whether the product is available elsewhere [25]

The guidance offered by these codes is limited. Neither provides a clear definition of, or restriction on, the types of products chiropractors may choose to sell, leaving the interpretation and resulting choice up to individual practitioners. At a minimum, products should be related to the practitioner's area of expertise. However, there is debate within the profession as to where the boundaries of this expertise lie, as illustrated by the comments provided for this study.

Both the provincial and national codes stop short of requiring that a scientific evidence base exist for products sold from offices, recommending instead that subjective review is sufficient. Although some respondents cited reviews of scientific evidence, others referenced manufacturer's data or simply stated they promoted things they used/liked or knew worked. The widely recognized lack of scientific evidence and uneven regulatory oversight for many health products [26-28], mean that reliance on an individual practitioner's opinion or preference may leave consumers further at risk. Others have proposed that members of their profession establish a board of peers charged with the review of evidence and approval of products for in-office dispensing based on established criteria [14].

The Canadian Code makes reference to product cost, arguably the issue at the centre of the potential conflict of interest. The standard recommended is "fair market price" which may be interpreted as what the market or, more accurately, consumers will bear, and is inconsistent with no profit-taking or cost-recovery transactions, leaving room for financial exploitation. While many respondents spoke of products retailing at cost, variation in product pricing was clear leaving consumers with inconsistency in practice standards. The provincial code is silent with respect to product pricing.

### Limitations

Despite the number of comments and the range of opinions expressed, the findings arising from the comments volunteered are not necessarily reflected of the chiropractic profession as a whole and may not generalize to practitioners in other areas, or to those who chose not to respond.

## Conclusion

Product sales in chiropractic appear well-established both provincially and nationally in Canada. As noted by some respondents, ethics and integrity are individual attributes. While this may be true, the actions of individual practitioners may affect public perception of the profession as a whole. To facilitate the integrity of professional judgment and to protect patient best interests, clearly articulated standards and consistency in practice are paramount.

The comments provided by the respondents suggest that existing guidelines may not be sufficiently detailed or stringent to adequately guide practice, or possibly that practitioners are simply not aware of these standards. It does not appear as though the profession has monitoring mechanism in place to assess compliance and enforce standards although several respondents suggested this would be a worthwhile undertaking on the part of the governing body. It would be worthwhile to systematically investigate how often complaints are received in this area by the provincial colleges or national association, and to determine whether disciplinary action has been taken.

Future research gathering data from a national sample of practitioners and examining patient perspectives will further inform the profession.

## Competing interests

The authors declare that they have no competing interests.

## Authors' contributions

SP designed and administered the study, conducted the preliminary data analysis and drafted the manuscript. JG contributed to the study design, data analysis and manuscript review. DGM contributed to the study design, data analysis and manuscript review. All authors read and approved the final manuscript.
